# Refractory Ventricular Fibrillation Treated with Double Simultaneous Defibrillation: Pilot Study

**DOI:** 10.1155/2020/5470912

**Published:** 2020-05-27

**Authors:** Hee Eun Kim, Kui Ja Lee, You Hwan Jo, Jae Hyuk Lee, Yu Jin Kim, Joong Hee Kim, Dong Keon Lee, Dong Won Kim, Seung Min Park, Young Taeck Oh

**Affiliations:** ^1^Department of Emergency Medicine, Seoul National University Bundang Hospital, Seongnam, Gyeonggi 13620, Republic of Korea; ^2^Department of Emergency Medical Services, Kyungdong University, Wonju, Gangwon 26495, Republic of Korea; ^3^Department of Emergency Medicine, Chuncheon Sacred Heart Hospital, Hallym University College of Medicine, Chuncheon, Gangwon 24253, Republic of Korea

## Abstract

**Introduction:**

Refractory shockable rhythm has a high mortality rate and poor neurological outcome. Treatments for refractory shockable rhythm presenting after defibrillation and medical treatment are not definite. We conducted research on the application of double simultaneous defibrillation (DSiD) for refractory shockable rhythms.

**Methods:**

This is a retrospective pilot study performed using medical records from 1 January 2016 to 31 December 2017. The prephase was from January to December 2016. The post-phase was from January to December 2017. During the prephase, we conducted conventional defibrillation with one defibrillator, and during the post-phase, we conducted DSiD using two defibrillators. Primary outcome was survival to hospital discharge. Secondary outcomes included survival to hospital admission and good neurological outcome at 12 months. Statistical analysis was conducted using Fisher's exact test. Data were regarded statistically significant when *p* < 0.05.

**Result:**

A total of 38 patients were included. Twenty-one patients underwent conventional defibrillation, and 17 underwent DSiD. The DSiD group had a higher survival to admission rate (14/17 (82.4%) vs. 6/21 (28.6%), *p*=0.001) and showed a trend for higher survival to discharge (7/17 (41.2%) vs. 3/21 (14.3%), *p*=0.078). Good neurological outcome at 12 months of the DSiD group was higher than that of the conventional defibrillation group, but the difference was not statistically significant (5/17 (29.4%) vs 2/21 (9.5%), *p*=0.207).

**Conclusion:**

In patients with refractory shockable rhythms, DSiD has increased survival to hospital admission and a trend of increased survival to hospital discharge. However, DSiD did not improve neurological outcome at 12 months.

## 1. Introduction

Survival rates for prehospital cardiac arrest are affected by factors such as bystander cardiopulmonaryresuscitation (CPR), witness of arrest, initial cardiac rhythm, and bystander CPR before emergency medical system (EMS) arrival [1]. Cardiac rhythms that require defibrillation (ventricular fibrillation/pulseless ventricular tachycardia, VF/pVT) show higher rates of survival than those that do not [2–4]. Current guidelines recommend rapid defibrillation for cardiac rhythms that require defibrillation [5, 6]. However, optimal treatment for refractory shockable rhythms remains unclear. The 2015 American Heart Association (AHA) and the European Resuscitation Council (ERC) guidelines recommend defibrillating and administering amiodarone for refractory shockable rhythms [3, 5]. Patients with shockable rhythm at the prehospital stage have a survival rate of 21.4–29.3% [7]. Patients with refractory shockable rhythm show a survival rate of 8.2% [8].

Recent studies exhibit diverse results regarding the treatment of patients with refractory shockable rhythms [9]. In one study, beta-adrenergic antagonists were recommended for refractory ventricular fibrillation (RVF) patients [10]. Double defibrillation has been recommended to increase survival rates for such patients [7, 11, 12]. Double defibrillation is a method where two sets of defibrillator pads are applied and two “simultaneous” or “sequential” shocks are delivered. It is easy to apply and can be used in the prehospital stage.

In this pilot study, we aim to determine whether the application of double defibrillation improves the rate of survival to hospital discharge, survival to hospital admission, and neurological outcome at 12 months in refractory VF/pVT patients.

## 2. Materials and Methods

### 2.1. Design and Setting

This is a retrospective pilot study. We obtained medical records of two regional Emergency Medical Centers from 1 January 2016 to 31 December 2017. The population of the study area is 849,992 and covers an area of 1,175.31 km^2^. The annual number of patients visiting the two emergency centers is approximately 100,000. The study was approved by the Hallym University Chuncheon Sacred Heart Hospital Institutional Review Board (CHUNCHEON 2018-10-011–003).

All cardiac arrest patients received advanced cardiopulmonary life support (ACLS) protocol according to 2015 AHA guidelines and received high quality ACLS by emergency medical personnel with ACLS provider certifications. Before an advanced airway was placed, chest compression and manual ventilation were carried out at a ratio of 30 : 2. In the prehospital stage, an i-gel supraglottic airway device is inserted by the EMS under medical direction. After arrival at the emergency department (ED), i-gel was changed to endotracheal tubes by a physician. After an advanced airway was placed, 2-minute cycles of compressions were given, and one breath was given every 6 seconds. One mg of epinephrine was given every 4 minutes until CPR was terminated. To patients receiving three defibrillation attempts without successful defibrillation, 300 mg of amiodarone was administered intravenously. If a shockable rhythm persisted, another 150 mg of amiodarone was administered. Chart review was carried out by two AHA ACLS instructors (HEK and KJL) and cross-checked by other two AHA ACLS instructors (DKL and YTO).

**Figure 1:**
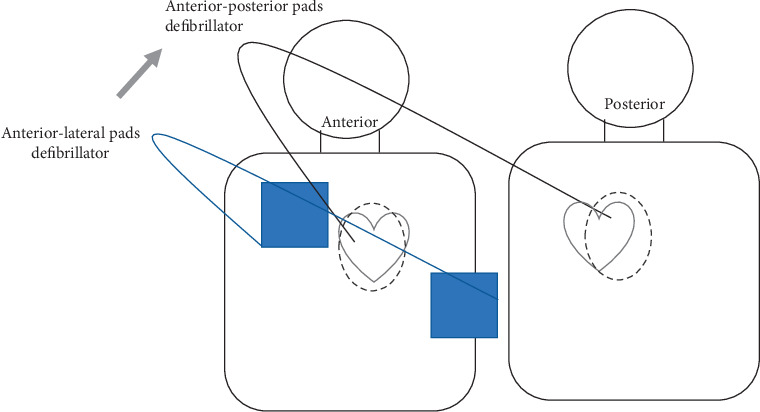
Double simultaneous defibrillation pad position.

At the prehospital stage, defibrillation was carried out with biphasic waveforms at 150J using anterior-lateral pads. All patients arriving at the ED were treated with a HeartStart MRx defibrillator (Philips Medical Systems, Andover, Massachusetts), a biphasic waveform defibrillator. Defibrillation with 200J was applied for in-hospital conventional defibrillation using anterior-lateral pads. Two defibrillators were used for double simultaneous defibrillation (DSiD). Anterior-lateral pads were attached first, and then anterior-posterior pads were attached using the second defibrillator machine. Pads were arranged so that they do not overlap (see Figure 1).

DSiD was applied by one person pressing both buttons simultaneously. Energy through each vector was set to 200J. To minimize time to DSiD, the number of times of conventional defibrillation was obtained through EMS, and if the patient received more than 3 defibrillations prior to arriving at the hospital, DSiD was applied immediately.

### 2.2. Study Population

Any cardiac arrest patient over 18 years of age with an initial shockable rhythm was included. Refractory shockable rhythm was defined as those that did not achieve return of spontaneous circulation (ROSC) after 3 cycles of defibrillation and 10 minutes of CPR. Exclusion criteria included (1) severe head trauma, (2) active bleeding, (3) severe sepsis, (4) terminal stage of malignancy, (5) severe neurological deficits, (6) initial nonshockable rhythm (asystole and pulseless electrical activity), (7) noncardiac origin causes, or (8) ROSC before 3 cycles of defibrillation.

We categorized the study population into prephase and post-phase according to the study period. Prephase consisted of patients who received conventional defibrillation from 1 January 2016 to 31 December 2016. Post-phase consisted of patients who received DSiD from 1 January 2017 to 31 December 2017.

### 2.3. Measurement of Variables

We analyzed the data by reviewing medical records. Prehospital data were acquired through patient care records provided by the EMS. The primary outcome of this study was survival to hospital discharge. Secondary outcomes were survival to hospital admission and good neurological outcome at 12 months. Neurological outcome was measured using the Glasgow–Pittsburgh cerebral performance category (CPC). CPC 1-2 were considered good neurological outcomes; CPC 3–5 were defined as poor neurological outcomes. In addition, age, sex, witness by laypersons, bystander CPR, time from call to EMS arrival, total prehospital/ED CPR time, total CPR time, number of prehospital/ED defibrillations attempted and joules, number of defibrillations total attempted and total joules, drugs and doses used, and clinical outcomes were reviewed, retrospectively.

### 2.4. Statistical Analysis

Continuous variables were assessed with the Shapiro–Wilk test for normality. Because no continuous variable followed a normal distribution, the median and interquartile range (IQR) were calculated. The Mann–Whitney *U* test was applied to evaluate the two groups. For nominal variables, Fisher's exact test was applied because expected variable was less than 5. Data were regarded statistically significant when *p* < 0.05.

## 3. Result

**Figure 2:**
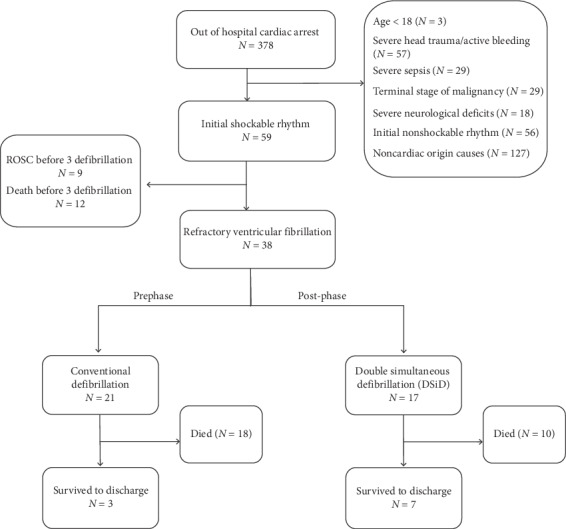
Flow chart of patient selection.

From January 2016 to December 2017, a total of 378 out of hospital cardiac arrest (OHCA) patients were reviewed. Of these patients, 59 had a shockable initial rhythm. Out of these 59 patients, patients that achieved ROSC or expired before 3 cycles of defibrillation were excluded. Of the remaining 38 patients, 21 patients received conventional defibrillation during the prephase and 17 patients received DSiD during the post-phase (see Figure 2).

The number of witnessed arrests in the DSiD group and in the conventional defibrillation group was similar (82.4% vs. 85.7%, *p*=1.000). Bystander CPR was delivered to 42.9% of the conventional defibrillation group and to 58.8% of the DSiD group (10 (58.8%) vs. 9 (42.9%), *p*=0.515, not significant.) (see Table 1).

The number of defibrillations of the DSiD group and the conventional defibrillation group did not statistically significantly differ (7 (IQR; 6–10) vs. 7 (IQR; 7–9.5), *p*=0.750). However, the total defibrillation joules delivered were higher in the DSiD group (1650 (IQR; 1375–2175) vs. 1200 (IQR; 1200–1675), *p*=0.026) (see Table 2). Total prehospital time in the DSiD group was 26 minutes; in the conventional defibrillation group, it was 21.5 minutes (26 (IQR; 22–29) vs. 21.5 (IQR; 15–30.7), *p*=0.347) (see Table 2).

Survival to hospital admission was higher in the DSiD group than the conventional defibrillation group (82.4% vs. 28.6%, *p*=0.001) (see Table 3). The survival to hospital discharge rate of the DSiD group (7/17) was higher than that of the conventional group (3/21), exhibiting a trend towards statistical significance (41.2% vs. 14.3%, *p*=0.078) (see Table 3). Good neurological outcome, both at discharge and at 6 and 12 months, in the DSiD group (5 patients, 29.4%) was higher than that of the conventional defibrillation group (2 patients, 9.5%), but was not statistically significant (*p*=0.207). Of those who survived to discharge, targeted temperature management (TTM) was applied to 7 patients in the DSiD group and 3 patients in the conventional defibrillator group.

## 4. Discussion

In our study, the DSiD group showed a trend for higher survival to hospital discharge compared to the conventional defibrillation group (41.2% vs 14.3%, *p*=0.078). In a study performed by Ross et al. [13], the survival to hospital discharge rate for DSiD patients was 4 out of 50 (8%) and those with conventional defibrillation was 33 out of 229 (14.4%). In Ross et al.'s study, survival to hospital discharge was not statistically significant different between DSiD and non-DSiD patients.

Double defibrillation can be categorized into sequential or simultaneous [14]. However, there is confusion in the use of sequential or simultaneous terminology in terms of applying double defibrillation. In past studies, many studies have been carried out regarding application of double defibrillation. Some described double defibrillation as double sequential but with doubtful details of method (with descriptions such as “as sequential as possible or near simultaneous”) [11, 15, 16], some calling their method “sequential” but actually describing simultaneous shocks [17], and some with clearly described sequential shocks [18, 19] or both sequential and simultaneous shocks [20]. Different methods of double defibrillation show a wide range of results. Research about DSiD (as in this study) shows various results [12, 13, 21]. In the research studies by Gerstein et al. [12] and Ross et al. [13], DSiD did not affect survival rate. In the study by Leacock et al. [21], DSiD did affect survival. Recently, Taylor et al. [22] reported in an animal study that “overlapping” shocks show the highest success rate in the porcine model. In this study, survival to discharge showed no statistical difference between the two groups, but showed a tendency to show higher survival in the DSiD group. This study may be underpowered because of the small sample size, but a better result is expected in further study. However, in our study, all survival to discharge patients went through TTM. Application of TTM affects survival rates and neurological outcomes in cardiac arrest patients [23]. In our study , there were no statistically significant differences between the DSiD group and the conventional defibrillation group in terms of TTM. Also, survival to hospital admission was evaluated independently of TTM.

In our study, survival to hospital admission of the DSiD group was 82.4% (14/15), and it was higher than that of the conventional defibrillation group (28.6% (6/21)) (*p*=0.001). In contrast, in a study by Ross et al. [13], the survival to hospital admission of the dual defibrillation was not statistically significant compared to the single defibrillation (32% (16/50) vs 37.6% (86/229), *p*=0.74). Ross et al.'s study was conducted at the prehospital stage, while our study was conducted at the ED. Also, there was a statistical difference in witnessed arrest and bystander CPR between the DSiD and conventional defibrillation groups. Our study showed the difference between the two methods compared to Ross et al. because there was no difference between the two groups in terms of witnessed arrest and bystander CPR.

Double defibrillation is a novel approach to treat refractory shockable rhythm. Its mechanism is not yet known. One hypothesis is that it decreases the defibrillation threshold of the myocardium [20, 24, 25]. In an animal study, two sequential shocks decreased the total energy and peak voltage to the myocardium [15]. This study suggests that the anterior-posterior pad position increased intracardiac electric current flow by transmitting an adequate amount of electrical current to depolarize the myocardium [16]. Another possible explanation for the mechanism of double defibrillation is that the two sequential shocks create two vectors that depolarize more myocardium than conventional defibrillation [19, 26]. In other case studies, higher rate of ROSC and complete neurologic recovery was shown in double defibrillation patients [15, 17, 21, 27]. On the contrary, in the study by Ross et al. [13], good neurological outcome (CPC 1 or 2) of the conventional defibrillation was 11.4% and that of the DSiD group was 6% without statistical significance. Neurological outcome at 12 months in our study also did not show statistical significance.

There are several limitations to our study. First of all, our sample size is small. Refractory shockable rhythms are rare. Despite the fact that the study sample was retrieved from two medical centers, the sample size is relatively small to represent the entire patient population. However, this study is not limited to a simple case report and shows the prognosis of such patients. Second of all, in the study, all patients surviving to discharge went through TTM. Other postcardiac arrest care, such as percutaneous coronary intervention and extracorporeal membrane oxygenation, were not taken into consideration. Third of all, the time to DSiD application was not measured. Recent studies showed early double defibrillation related to a higher survival rate [11, 28]. A follow-up study is required for the effects of early DSiD. Fourth of all, there is a limit to the analysis of medical records due to the limited information provided by the patient care records. Lastly, due to the design of the study, other uncontrolled confounding variables may have affected our results. We intend to compensate such shortcomings in subsequent studies.

## 5. Conclusion

DSiD in refractory shockable rhythm patients was shown to improve survival to hospital admission and a trend of increased survival to hospital discharge. However, differences in neurologic outcomes were not statistically significant.

## Figures and Tables

**Table 1:** Comparisons of baseline characteristics between the DSiD-treated and conventionally treated groups.

	DSiD (*N* = 17)	No DSiD (*N* = 21)	*p value*
Age, median (range), yr	60 (18–83)	65 (18–93)	0.487
Male, *n* (%)	14 (82.4)	17 (81.0)	1.000
Witnessed by laypersons, *n* (%)	14 (82.4)	18 (85.7)	1.000
Bystander CPR, *n* (%)	10 (58.8)	9 (42.9)	0.515
Time from call to EMS arrival, minutes;median (IQR)	8.5 (6.8–11)	7 (4–10)	0.246

CPR: cardiopulmonary resuscitation; EMS: emergency medical system.

**Table 2:** Comparisons of treatment between the DSiD-treated and conventionally treated groups.

		DSiD (*N* = 17)	No DSiD (*N* = 21)	*p value*
Prehospital defibrillation attempts; median (IQR)		4 (2.5–6)	3 (3–4)	0.095
Prehospital defibrillation joules; median (IQR)		600 (375–900)	450 (450–600)	0.095
ED defibrillation attempts; median (IQR)		3 (2.5–4)	4 (3–6.5)	0.045
ED defibrillation joules; median (IQR)		950 (750–1350)	750 (550–1250)	0.220
Total defibrillation attempts; median (IQR)		7 (6–10)	7 (7–9.5)	0.750
Total defibrillation joules; median (IQR)		1650 (1375–2175)	1200 (1200–1675)	0.026
Total prehospital time, minutes; median (IQR)		26 (22–29)	21.5 (15–30.7)	0.347
Total ED CPR time, minutes; median (IQR)		23 (4.5–38.5)	31 (24.5–35)	0.128
Total CPR time, minutes; median (IQR)		48 (33–56)	51 (44.2–60.7)	0.564
Adrenaline, mg; median (IQR)		6 (2.5–10)	8 (6–9)	0.161
Amiodarone, mg; median (IQR)		450 (375–450)	450 (450–450)	0.337
Sodium bicarbonate (mEq), median (IQR)		3 (3–3)	4 (3–5)	0.333
Targeted temperature management, *n* (%)		10 (58.8)	6 (28.6)	0.099

ED: emergency department; CPR; cardiopulmonary resuscitation.

**Table 3:** Comparisons of outcome between the DSiD-treated and conventionally treated groups.

	DSiD (*N* = 17)	No DSiD (*N* = 21)	*p value*
Survival to hospital admission, *n* (%)	14 (82.4)	6 (28.6)	0.001
Survival to hospital discharge, *n* (%)	7 (41.2)	3 (14.3)	0.078
Good neurologic outcome (CPC 1 or 2) at 12 months, *n* (%)	5 (29.4)	2 (9.5)	0.207

CPC: cerebral performance category.

## Data Availability

The data used to support the findings of this study have not been made available because it is not public data.
